# Issues in the surgical management of skin squamous cell cancers in albinos—experience of two surgical oncology units in Burkina Faso

**DOI:** 10.1186/s12957-023-03217-0

**Published:** 2023-10-13

**Authors:** Nayi Zongo, Adeline R. Djiguemde, Parateyandé Bonaventure Yameogo, Abdoul Halim Bagué, Sidy Ka, Bangaly Traoré, Niamba Pascal, Ahmadou Dem

**Affiliations:** 1 Digestive and General Surgery, Yalgado Ouedraogo Teaching Hospital Ouagadougou, Joseph Ki-Zerbo University, 03 BP 7021 Ouagadougou, Burkina Faso; 2 Joliot Curie Institute of Dakar (Senegal), Cheikh Anta Diop University of Dakar, 10700 Dakar, Senegal; 3 Surgical Oncology Unit, Hospital Donka, Conakry, Guinée

**Keywords:** Albinos, Skin cancer, Multifocal, Lumpectomy, Skin flaps

## Abstract

**Background and objectives:**

Skin cancers in albinos are frequent in sunny countries. The surgeon plays a crucial role in their treatment. The objective was to describe the challenges of surgical management of skin cancer in albinos.

**Methods:**

Retrospective, descriptive, and multicenter study on skin cancer surgery in albinos performed over the past 14 years in Ouagadougou. We were interested in surgery indications, techniques, and results. Survival was assessed using the Kaplan–Meier method. Comparisons of proportions were made by Student’s *t*-test.

**Results:**

The cancers were multiple synchronous in 41.3%. We identified 46 albinos with 71 skin cancers. Surgery was performed in 93%. Lesions were located on the back, upper limbs, and head and face in 40.9%, 30.3%, and 16.7%, respectively. Precancerous lesions were treated concomitantly in 23.6%. The surgery consisted of a lumpectomy. Direct suturing and mobilization of flaps allowed skin coverage in 17.9% and 34.3%, respectively. Lymph node dissection was associated with the limbs in 73.1% of localizations. The average number of lymph nodes removed was 11, with extremes of 7 and 14. Node invasion was noted in 16 out of 19 cases. The resection margins were invaded in 7.5% and required surgical revision. Recurrences were noted in 8.9% of cases. Overall 2-year survival rate was 55.8%.

**Conclusions:**

Surgery must meet the triple challenge of treating single or multiple synchronous cancers, precancerous lesions, and allowing good healing. Early diagnosis would reduce the rate of secondary healing and improve survival. The absence of extemporaneous histology and the large size of the tumors associated with the delay in diagnosis meant that surgery, whenever possible, was limited to wide and deep resection, to ensure healthy margins.

## Highlights

The surgeon plays a crucial role in the management of skin cancers in albinos. He resects the cancers. He must also ensure skin coverage during the same operating time or at a different time after large resections, using oncoplastic techniques if necessary. He participates in the prevention of skin cancers in this at-risk group by detecting and removing precancerous lesions. In collaboration with dermatologists and oncologists, he participates in the planning of medical treatments and radiotherapy. He also helps to raise awareness among albinos of the risks and means of protection against skin cancers.

## Introduction

Skin cancers are rare in black Africa [1, 2]. However, albinos who live in these very sunny countries constitute a population at risk, due to their melanin deficiency [3–5]. They have a significant proportion of skin cancers. Albinos are generally poor, forced to live on farming work under the blazing sun in the African context [6, 7]. This is why the exposed parts of the body are the main ramparts of albino skin cancers [6, 8, 9]. The prevalence of skin cancer in albino populations varies from country to country, ranging from 2.07 to 26% [10, 11]. These cancers are particular, as they occur in relatively young subjects, are multiple, synchronous or metachronous, coexist with precancerous lesions, and cause death before the age of 50 [12]. Surgery is the main curative treatment for skin cancer in albinos [7, 13]. It is based on the principles of Mohs micrographic surgery for countries where there is extemporaneous examination [13–15]. Wide and deep resection followed by direct closure or other healing processes are described in many countries where extemporaneous examination is not possible [7, 12].

In Burkina Faso, estimated at 3000–5000, albinos regularly die and almost exclusively from skin cancers. Studies on skin cancers exist in general. The multifocality of albino skin cancers and the frequent coexistence of precancerous lesions make surgery special and sometimes difficult. However, no study has been specifically devoted to the surgical management of these cancers in the West African context. This is why we undertook this work with the aim of describing the indications, procedures, and peculiarities of skin cancer surgery in this group.

## Patients and methods

### Type and period of study

This was a cross-sectional survey that took place between 1st January, 2013 and 31st December, 2022. It concerned all albinos being followed for skin squamous cell cancers. We also questioned the presidents of albino associations to register what they think of skin cancers.

### Study site

The study was conducted in two surgical oncology units in Ouagadougou, Burkina Faso. These units are reference health structures for the treatment of skin cancers. In this country, albinos are poor and do not have health insurance.

### Study population

We were interested in all albinos followed for skin squamous cell cancers.

#### Inclusion criteria

We included all patients with a clinical file containing sufficient information on diagnostic aspects and surgical management.

#### Exclusion criteria

We excluded from the study albinos followed for skin cancer but not operated on due to non-resectability. We excluded from the study files without sufficient information on surgical procedures.

### Data collection procedures

Patient clinical records, consultation registers, and operating reports constituted our data sources. For each patient, we looked at the sociodemographic data (age, sex, place of residence), histological type, indications for surgery, surgical procedures performed, and survival data. Concerning the survey with presidents of albino associations, we first contacted them and got their consent. There are two albino associations run by albinos themselves, which advocate for their welfare. These associations have all been existing for more than 5 years and are very active on the ground. We interviewed the presidents of the two well-known albino associations in the country. We met them and asked our questions directly. The questions focused on the estimated number of albinos living in Burkina Faso and the prevalence of skin cancers in the albino population. They gave their views on the attitudes they teach their members and the means of protection they use against skin cancers. Questions were also asked about the measures they felt albinos and presidents of albino associations should take to prevent albino deaths from skin cancer. These presidents were not paid to take part in this survey.

### Data analysis management and strategy

Excel 2010 and R software in version 4.1.0 were used for the analysis. We appreciated the gestures made according to the size and the topography. We classified the tumors according to the UICC TNM 2018 classification of skin squamous cell cancers. T1 represents cancers smaller than 2 cm. T2 represents carcinomas of 2 to 4 cm. Tumors are classified as T3 when the size is greater than 4 cm or in cases of deep involvement and T4 when there is significant bone or medullary involvement. Lymph node involvement is classified as N1, N2, and N3 respectively in cases of lymph node involvement of less than 3 cm, more than 3 cm, or multiple lymph nodes of more than 6 cm. Stages I and II refer to localized carcinomas. Stages III and IVA refer to locally advanced cancers and stage IVB to metastatic carcinomas. Comparisons of proportions were made using the Student’s *t*-test. Survival was assessed using the Kaplan–Meier method.

### Ethical aspects

The study was authorized by the management of the hospitals and the heads of the surgery department concerned. The use of illustrative photos was authorized by the patients. Anonymity was respected for all patients.

## Results

### General data

**Fig. 1:**
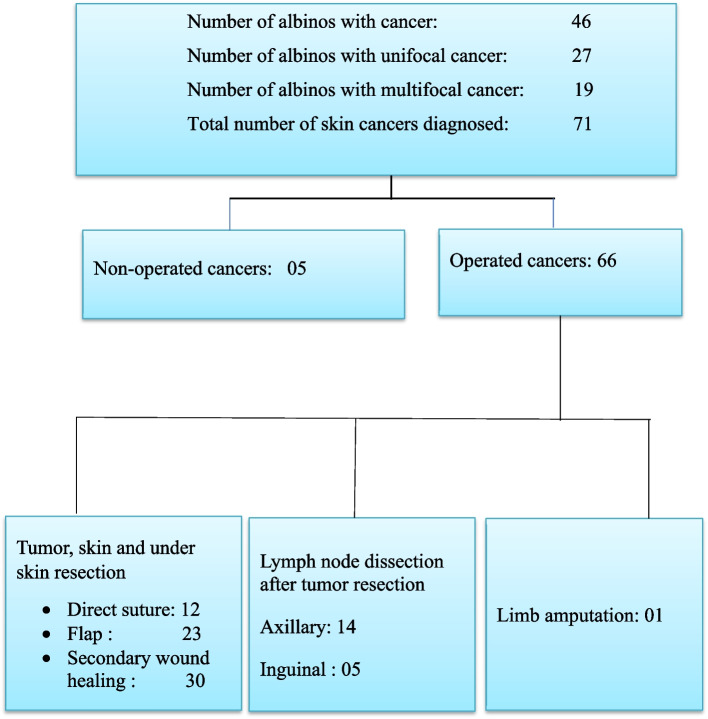
Flow chart

Over 14 years, we have examined 139 albinos including 57 during a surgical consultation and 82 during a survey on the attitudes, knowledge, and practices of albinos towards skin cancer, carried out in 2016. Forty-six (46) were carriers of skin cancer (Fig. 1). Albinos presented single cancers in 27 cases (58.7%) and multifocal cancers in 19 cases (41.3%) (Table 1, Fig. 1). We noted a total number of 71 cancers in these 46 albinos (Fig. 1). The average age of the patients was 36.3 years ± 9.2. They were women in 66% and men in 34% of cases, i.e., a sex ratio of 0.5 (Table 1). The lesions were localized on the parts exposed to sunrays, in particular the back, the upper limbs, and the head and the face in 40.9%, 30.3%, and 16.7%, respectively. They were ulcerated (100%), hemorrhagic (43.9%), and necrotic (18.2%) (Table 1). The average size of the lesions was 9.2 ± 7.2 cm (Table 1). The average consultation time was 18.7 ± 8.3 months. Three (4.5%) patients had a tumor classified as stage I (Table 1). At diagnosis, the cancers were already metastatic in 5.5% of cases.

**Table 1:** General data of albinos operated for skin cancer (*n* = 66)

Items	Categories	Number	%
Gender (*n* = 46)	Male	16	34.8
	Female	30	65.2
Age (*n* = 46)	15–25	12	26
	25–35	28	60.9
	35–45	5	10.9
	45–55	1	2.2
Origin (*n* = 46)	Urban environment	5	10.9
	Rural areas	41	89.1
Profession (*n* = 46)	Civil servant	1	2.2
	Cultivator	15	32.6
	House wife	23	50
	Trader	3	6.5
	Pupil/student	4	8.7
Socio-economic level (*n* = 46)	Bottom	38	82.6
	Medium	6	13
	Top	2	4.3
Macroscopic appearance of lesions (*n* = 66)	Ulcerated	66	100
	Hemorrhagic	29	43.9
	Necrotic	12	18.2
Topographic of lesions (*n* = 66)	Lower limb	6	9.1
	Upper limb	20	30.3
	Face and neck	11	16.7
	Back	27	40.9
	Chest	3	4.5
Number of lesions per patient (*n* = 46)	Unique	27	58.7
	Two	12	26.1
	More than two	7	15.2
Primary or recurrent (*n* = 66)	Primary	56	84.8
	Recurring	10	15.2
Stages (*n* = 66)	I (T1N0)	3	4.5
	II (T2N0)	16	24.2
	III (T3N0)	30	45.5
	IVA (all T; all N1, N2, N3; M0)	17	25.8

### Results of the survey with presidents of associations of albinos

“We are around 3000-5000 albinos in Burkina Faso. Albinos are under 50 in more than 98% of cases. Albinos do not have health insurance and their families are mostly poor. Since 2013 all albino deaths have been from skin cancer. They all died before their fiftieth anniversary. We work together with oncologists and dermatologists, to protect albinos by raising awareness on the dangers of sunrays. With the help of our partners, we are working to ensure that many albinos as possible have means of protection against sunrays. These means include hats, long-sleeved clothes and creams. We advise them to stay in the shade between 10 a.m. and 4 p.m., which is the time interval when the sun is at its strongest. We are also working so that albinos can have a normal schooling, can work in offices and give up work in the fields where they are constantly exposed to the blazing sun. We organize screening campaigns for precancerous lesions and assist albinos in the management of these lesions.”

### Surgery for skin cancer in the albinos

**Fig. 2:**
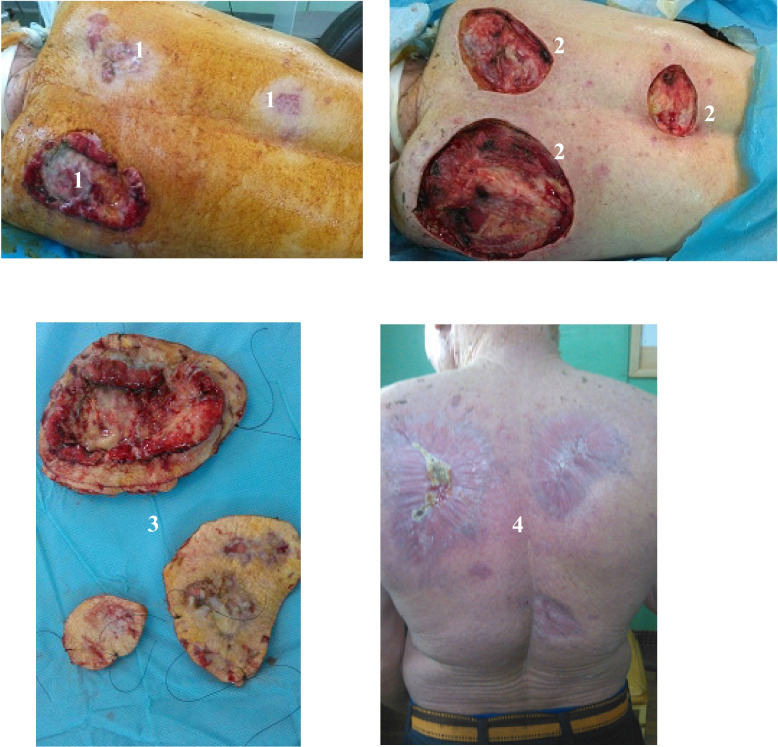
Multiple squamous cell carcinoma of the back of an albino treated by resection + secondary wound healing. 1: Multifocal tumor of the back of the albino. 2: Operative wounds after wide resection. 3: Oriented operative specimens for pathological anatomy. 4: Scars after 4 months of secondary healing

**Fig. 3:**
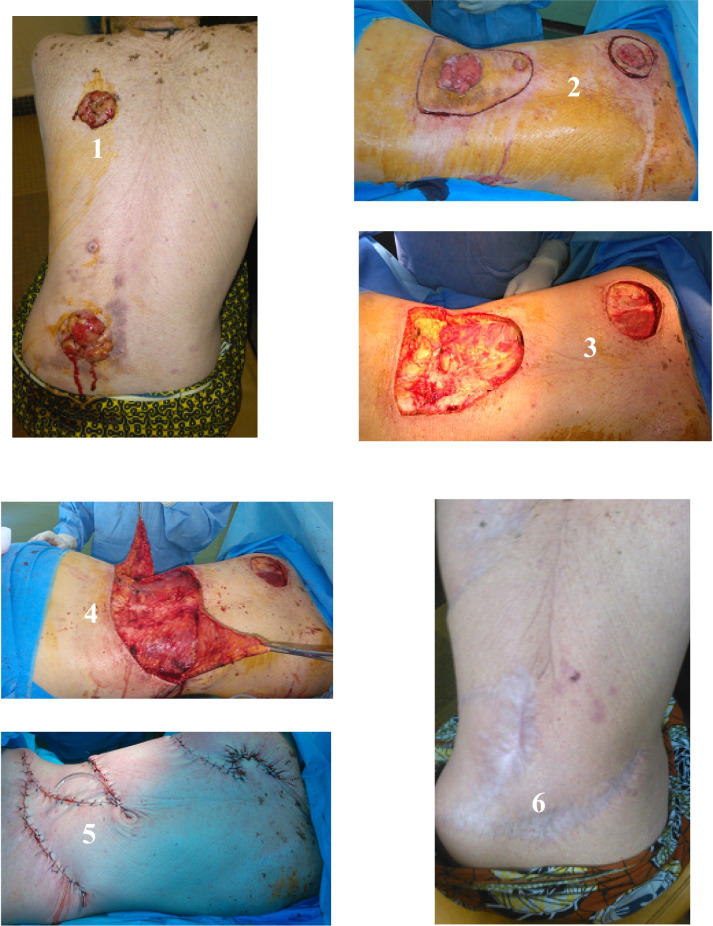
Multifocal squamous cell carcinoma of the back of an albino treated by resection and O to Z plasty. 1: Multifocal squamous cell carcinoma of the back. 2: Cut. 3: Surgical wounds. 4: Mobilization of the flaps. 5: O to Z plasty. 6: Operative scar with a follow-up of 1 year

Dermatologists, oncologists, and surgeons also work closely together to monitor patients under treatment. We opted for surgical abstention in 5.5% of cases, due to metastasis and in 1.4% of cases due to unresectability. Ninety-three percent of the 71 skin squamous cell cancers in albinos were operated on. We performed multiple resections in 40.4% of patients. These multiple resections carry in the same operating time two, three, or even more cancerous lesions (Figs. 2 and 3).

We also resected 17 precancerous lesions concomitantly with confirmed cancers. It was essentially actinic keratosis in 11 cases and chronic ulcerations in 6 cases. The lack of extemporaneous histological examination in our context required wide resections with margins of 2 to 4 cm and also deep resections that removed the aponeurosis in 71.6% of cases. Concerning the surgical procedures, we performed lumpectomy followed by skin coverage by direct suture in 18.2% of operated cancers. A wide lumpectomy followed by secondary healing was the surgical procedure in 45.5% (Fig. 2, Table 2). Excision of the tumor left a major defect in place, requiring the mobilization of skin flaps in 34.8% of cases (Table 2, Fig. 3). The resection margins were invaded in 7.5% of cases. Revision surgery was performed in all these cases. In 73.3% (19 cases) of tumors localized to the limbs, axillary or inguinal lymph node dissection was associated. The average number of lymph nodes removed was 11, with extremes of 7 and 14. Node invasion was noted in 16 out of 19 cases.

**Table 2:** Distribution of operated patients according to tumor size, surgical procedure, excision margins, and evolution (*n* = 66)

Characteristics	Surgical procedure				Total
	Tumor resection + direct suture	Tumor resection + flap	Tumor resection + directed healing	Amputation of limb	
Topography					
Lower limb	2	0	4	0	6
Upper limb	4	7	8	1	20
Face and neck	1	3	7	0	11
Back	4	13	10	0	27
Chest	1	0	1	0	2
Tumor size					
< 2 cm	4	0	0	0	4
< 5 cm	8	8	0	0	16
> 5 cm	0	14	17	0	31
> 10 cm	0	1	14	1	16
State of excision margins					
R0	11	20	29	1	62
R1^a^	1	2	2	0	5
Evolution after surgery					
Local recurrence	0	1	3	0	4
Second cancer^b^	2	1	2	0	5
Metastasis^c^	0	2	3	1	6
Lost to follow-up	0	4	5	0	9
Complete remission	10	15	18	0	43
Total	12	23	30	1	66

R0: macroscopically and microscopically healthy margins

R1: macroscopically invaded

^a^All tumors with R1 resection were reoperated

^b^The patient developed another cancer during surveillance in another part of the body

^c^All metastatic patients died

### Adjuvant treatments

Radiotherapy is not available in Burkina Faso from 1st January 2013 to 31st December 2022. None of the albinos benefited from radiotherapy. It was indicated in 71.1% of cases. The indications for radiotherapy were a significant tumor size with deep infiltration, the presence of perineural sheathing, or the presence of perivascular sheathing in 37 cases (56.1%) and/or lymph node invasion in 16 cases (24.2%). Chemotherapy was indicated in 32.8% of cases. It was actually carried out in 12% of cases.

### Evolution

**Fig. 4:**
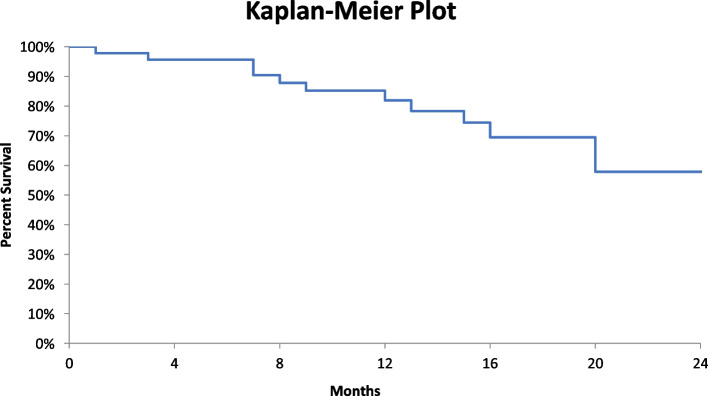
Survival curve for albinos operated on for skin cancer

The average healing times were 16 days ± 6, 23 days ± 7.3, and 72 days ± 22.7 for direct sutures, skin flaps, and secondary healing (*p* = 0.02), respectively. The tumor recurrence rate was 8.9% with an average onset time of 11 months (Table 2). Second cancers were noted in 7.5% of cases. We observed the appearance of metastases in 8.9% of these operated patients. After an average follow-up of 2 years, 17 patients, i.e., 23.6%, were lost to follow-up. Overall 2-year survival was 55.8% (Fig. 4).

## Discussion

There are few national data on the prevalence of skin cancers in the albino population. However, hospital studies show that the prevalence of skin cancers among people with albinism varies from one country to another. In Togo, Benin, Mali, Nigeria, and Brazil, for example, prevalence rates of 12.4%, 2.07%, 2.29%, 13.8%, and 26% have been reported, respectively [10, 11, 16–18]. Furthermore, according to the presidents of albino associations, all albinos who are inadequately protected in sunny countries like Burkina Faso end up dying from skin cancer. Albino cancers have the characteristic of being multifocal in the context of the sunniest countries of the planet [12, 13]. Observing the literature on tropical Africa, we noted that the proportion of synchronous multifocal cancers reached 36.7% in some series [12]. In our series, more than a quarter of albinos had at least two cancerous lesions on the trunk or on one of the limbs. This multifocality is a surgical challenge. However, it is above all the frequency of these cancers that is more disconcerting [6, 7, 13]. In black Africa, skin cancers are globally rare. However, their prevalence among albinos is high [12, 19–21]. That is why health education is so important. It should make it possible to inform albinos, their families, the general population, educators, and health professionals about the peculiarities of this genetic condition [22–25]. The presidents of albino associations questioned in this study insisted on the need for their protection against sunrays. This protection includes the wearing of long-sleeved clothes, hats, and the use of anti-sun creams [6, 9]. Dermatologists play an essential role in prevention through advice and in the detection of precancerous lesions through routine visits. They play a full part in the diagnostic process and in the indications for treatment. Dermatologists, oncologists, and surgeons also work closely together to monitor patients undergoing treatment. Dermatoscopy has improved the early detection of skin cancers [26, 27]. This allows less invasive surgery for small tumors and also reduces the cost of care. However, in our series, the cancers were so advanced that the characteristics of malignancy could be seen with the naked eye, on clinical examination by the oncologist or dermatologist. This explains why we did not use a dermatoscope.

In addition to prevention, treatments can help avoid deaths of albinos from skin cancer. Surgery is the mainstay of management [15]. It is the main curative treatment. The surgeon is faced with the multifocality of skin cancers in albino patients [12, 13]. In the literature, surgery is possible in 56.7 to 85.5% of cases [7, 12]. Ninety-three percent of our patients were operated on. The reasons for ineligibility for surgery were mainly the advanced stages, but also the financial difficulty of the patients. The surgeon must concomitantly ensure the resection of single or multiple lesions and the resection of precancerous lesions [24, 28]. Surgical treatment of skin cancer in albinos responds to the same principles as surgery for skin cancer in non-albinos [15, 29, 30]. In countries where there is extemporaneous histological examination, the size of the resection can be adapted to the tumor according to the Mohs micrographic technique [28, 30, 31]. It saves resection while remaining oncological [28, 30, 31]. This saving of skin increases the rate of direct suturing after lumpectomy and reduces postoperative complications [31]. In our series, as in other African series, the absence of extemporaneous histology and the large size of the tumors associated with the delay in diagnosis meant that surgery, whenever possible, was limited to wide and deep resection, to ensure healthy margins [12, 15]. The histology is generally obtained 3 weeks after the surgery and in the event of invaded margins, a reoperation is essential. This was done in 7.5% of our cases.

The corollary detailed diagnosis of large tumor size makes skin coverage by direct suture difficult [12, 13, 15]. This is why we performed a direct suture in only 18.2% of cases. Secondary healing is the source of many wound dressings, long healing times, increased treatment costs, and unsightly scars [13, 15]. Despite all these disadvantages, it was the option in nearly half of our operated patients and in ¼ of the cases in another West African series [12]. This shows the importance of increasing awareness for early diagnosis, but also of using other skin covering techniques when direct suturing is not possible [5, 32]. In the literature, there are many alternatives to secondary healing. These include musculocutaneous pedicled flap which require great technical skills, non-pediculated skin flaps that are easy to perform, even in a precarious situation, and immediate or delayed skin grafts [15, 33]. We opted for non-pediculated flaps, because of its ease of execution, its feasibility even in a precarious situation, and its satisfactory results [15]. Indeed, these flaps made it possible to carry out O to Z plasty. They allow rapid healing, reduction in the number of dressings and days of absence from work for the patient, and finally it gives a more beautiful scar than secondary healing [13, 15]. Above all the evolution of skin cancers in albinos is affected by delay in diagnosis and it is difficult to do skin coverage. Overall survival in our series was 55.8% at 2 years. It was 29.0% at 3 years in the Guinean series [12]. Albinos have the particularity of presenting second primary cancers in proportions reaching 7.5% of cases [12]. These second primary cancers can be avoided by using means of protection against sunrays. The presidents of albino associations interviewed recommend sun avoidance which consists of staying in the shade between 10 a.m. and 4 p.m., the time of day when the sun is blazing.

## The weaknesses of the study

The study certainly had limitations. The data collection was retrospective in a country without a computerized data management system. Furthermore, albinos living in their village feel often marginalized and go rarely to hospitals. They live in precarious conditions and have no easy access to diagnosis and care. This probably led to an underestimate of the number of cases. The number of lost from sight from the first year of follow-up was important and this occasioned data loss. The wound coverage techniques used in these specialized centers, performed by surgeons with good knowledge of oncology and surgery reconstruction, do not reflect what albinos get as surgery in peripheral structures. These limitations call for improved management of patient records, equitable diagnostic and therapeutic access for all albinos, and teaching of albino skin cancer surgery techniques to all surgeons, even those practicing in peripheral centers. This will not only improve survival rates, but also provide more complete and reliable data.

## Conclusion

Albino skin cancers are common. Surgery must meet the triple challenge of resecting cancers, precancerous lesions, and allowing good healing. Early diagnosis would reduce the rate of secondary healing and improve survival and quality of life. The absence of extemporaneous histology and the large size of the tumors associated with the delay in diagnosis meant that surgery, whenever possible, was limited to wide and deep resection, to ensure healthy margins. Primary prevention involves protection from sunrays. Secondary prevention involves the detection and correct treatment of precancerous lesions common in this group. The organizing of continuing education seminars on skin cancers, surgical techniques for skin cancers, and reconstructive surgery for surgeons would provide better care for patients. Also, a study including patients from several West African countries where albinos experience the same realities, with similar diagnostic and therapeutic pathways, could provide more information on albino cancer surgery by including a larger number of patients.

## Data Availability

The data used are available. Collection sheets containing the data used are available with the corresponding author. Patient files are available in the file rooms of the hospitals used for the study. These were the CHU Yalgado Ouedraogo and the Schiphra Protestant Hospital.
